# Life at the replication fork: A scientific and personal journey

**DOI:** 10.1016/j.jbc.2024.105658

**Published:** 2024-01-12

**Authors:** Charles S. McHenry

**Affiliations:** Department of Biochemistry, University of Colorado, Boulder, Colorado, USA

I was born on January 1, 1948, in Indianapolis, Indiana. My father was a particularly conscientious and hard-working railroad engineer who began his career with coal-fired steam engines. He was away a lot because hours were long and variable, and his job often required staying at the other end of the line. My mother had worked as a telephone operator earlier in her life but became a stay-at-home mom while my sister and I were being raised. My father had a mechanical bent and encouraged me along those lines, helping me with projects such as making telegraphs that I used to communicate with a neighbor boy with wires stretched over the driveway. Christmas gifts included microscopes, chemistry sets and the like, which kindled my initial interest in science.

For reasons stemming from my childhood that I won’t go into, when I started grade school, I recall being confused and disoriented. I had not developed emotionally to the point of being able to interact with the outside world effectively. I entered these years with a deep-seated sense of confusion, sense of shame and feeling I was not good enough—a theme that repeats within this article. By the third grade, I had learned to use a nascent drive for success to compensate for my perceived deficiencies. I learned that academic success provided an avenue for my being OK. I became ambitious early in life. I earned my Eagle Scout, and I was elected senior patrol leader at an age earlier than most, which gave me both the opportunity and the challenge of learning how to lead boys older than me.

## Undergraduate and graduate education

I went to Purdue University in 1966 on an academic scholarship awarded by the Evans Scholar Foundation, an arm of the Western Golf Association. To apply, one had to have caddied at one of their affiliated country clubs for 3 years. I learned about this opportunity early in high school and set my sights on it. Having my tuition and housing covered by this award allowed me to pay my own way through my undergraduate education with weekend and summer jobs. The Evans Scholars were housed together and had a fraternity-like setting except taking academics more seriously than most. I was elected President of the house during my junior year and became a member of Purdue’s interfraternity council. I pursued a chemistry major and became very interested in organic chemistry, doing undergraduate research in Henry Feuer’s lab. I was sure all too soon that this would be my future, even being brash enough to attempt to petition out of the biology course requirement. Fortunately, my request was denied. As an advanced student, I did not want to take a 101-level entry course and instead opted to take a graduate course that was offered for non-biologists. It was taught by David Hass, a senior fellow in Michael Rosenberg’s lab. Lectures were supplemented with reviews, monographs, and lectures from other faculty in the Biology Department. I was quickly seduced.

I had already started applying for graduate school. With my new-found interest in biochemistry, I modified my search for graduate schools to include a subset of chemistry departments with faculty interested in biochemical problems—ubiquitous now, but not in 1969. I accepted an offer from the Department of Chemistry at the University of California, Santa Barbara. I confess a desire to escape the Midwest and the attraction of a campus adjacent to the Pacific coast. I was drawn to the work of two senior professors—Tom Bruice and Bill Baker.

I started graduate school in 1970, taking a rigorous course taught by Tom Bruice during my first year on chemical mechanisms of enzymatic reactions, which emphasized model organic reactions. The course was rarely given, so it contained many senior students, including those from Tom’s lab. I wrote a required research proposal towards the end of the course on potential mechanisms for flavins in oxidation reactions. Tom told my undergraduate advisor it was the best in the class. This gave me a strong jump-start in confidence and in physical organic chemistry. I was tempted to join Tom’s lab, but I was attracted by interesting mechanistic projects with thymidylate synthase (TS) in Dan Santi’s lab. Students in Dan’s lab had conducted studies with model organic compounds that showed that uracil was susceptible to Michael additions at the 6-position and posited that methylation of dUMP to form dTMP proceeded by such a mechanism. For my PhD dissertation topic, Dan offered me a project using TS to test this mechanistic hypothesis. I eagerly accepted and, with Dan, chose a plan using the cancer chemotherapeutic drug, 5-fluoro uracil (FU) as a test. It was known that this compound was converted to 5-fluoro deoxyuridylate (FdUMP) within cells and that it bound tightly to TS, blocking DNA replication by depletion of dTTP.

Dan left on sabbatical to UCSF shortly after I started. In retrospect, this was a great opportunity to learn how to work without much supervision, developing confidence and independence in a research setting. Using an approach that involved pre-incubating FdUMP with TS, I initially failed to observe inhibition. But, upon addition of the cofactor 5,10-methylene tetrahydrofolate (CH_2_-FAH_4_), the enzyme was rapidly inactivated, even at a 1:1 ratio between FdUMP and TS active site. I was able to isolate complexes between FdUMP and TS that were not disrupted by urea denaturation of the enzyme. The uracil chromophore was lost during the reaction, consistent with the hypothesis that a nucleophile attacked FdUMP at the 6-position and that the reaction was irreversible because of fluorine at the 5-position (1). Completing this work during my first year in Dan’s lab gave me a sound foundation for the rest of my graduate career.

Dan’s sabbatical at UCSF resulted in an offer of a position there, which he accepted. The lab moved to UCSF in early 1972, and this opened a whole new world for me. The Biochemistry Department at UCSF under the leadership of Bill Rutter was in the exciting initial phase of transforming into an exceptionally strong center for molecular biology. Rutter’s lab had purified eukaryotic RNA polymerase and was interested in using it to explore gene regulation. Gordon Tomkins was establishing the basic features of steroid receptor action using rat liver hepatoma cells that were able to grow in significant quantity in tissue culture. Herb Boyer (later the co-founder of Genentech) was purifying *Eco* RI restriction endonuclease, showing it cut with ‘sticky’ ends that could hybridize to any DNA similarly cleaved. This led to one of the initial successes with molecular cloning.

I signed up for the inaugural year of a molecular biology course team-taught by Brian McCarthy, Howard Goodman, and Herb Boyer. All teaching was literature-based. The exams were all oral with one-to-one interaction with the full teaching team. If a student answered the basic questions correctly, they stepped up the challenge, providing a wonderful opportunity for growth and thinking on one’s feet and establishing relationships with key faculty. Brian became an informal co-advisor for me, helping me with postdoctoral choices and providing support early in my career. In addition, the early years presented additional heroes to me, among them Gordon Tomkins and Bruce Alberts. I did not know either well, but observing them carefully from afar, I was impressed by their free and original thinking and their lack of being tethered by dogma or the restraining conventions of society.

In my research, I exploited radioactive FdUMP to develop a quantitative assay for TS, working with both bacterial extracts and the cytosol of rat liver hepatoma cells (2). I also dug deeper into the mechanism of FdUMP’s inactivation of TS. I demonstrated a secondary isotope effect using 6-tritiated FdUMP, consistent with an sp^2^ to sp^3^ hybridization change (3). During the final months of my graduate work, I devised a home-made, semi-rapid, mix-and-quench approach to evaluate the kinetics of the overall reaction of TS at various temperatures. This allowed the kinetic and thermodynamic landscape of the reaction to be determined. This last chapter of my dissertation was not published until I was a full professor and was enhanced greatly by the analysis Ron Raines contributed as a postdoctoral fellow with Bill Rutter (4).

My graduate work showed that FdUMP action was dependent upon CH_2_-FAH_4_. It was common at the time in cancer treatment to combine FU with methotrexate, the latter inhibiting dihydrofolate reductase and reducing cellular levels of FAH_4_. Dan Santi and Dave Martin collaborated to show that indeed methotrexate decreased the cytotoxicity of FU in mouse leukemia cells in tissue culture (5). This led to the use of a folate analog, Leucovorin, in combination therapy with FU (6).

## Postdoctoral research at Stanford with Arthur Kornberg

From the molecular biology course I took at UCSF, I became interested in work from Arthur Kornberg’s lab, led by Randy Schekman and others, that had used phages that are dependent on the *Escherichia coli* (Eco) replication apparatus as an assay to identify and isolate 10 protein fractions that together can replicate φX174 phage DNA *in vitro* (7). This seemed like a fascinating opportunity to expand my enzymology skills. I applied to Arthur, and he immediately invited me to drive down the road to Stanford for an interview. Within a week, I had accepted a postdoctoral position in his lab in the Biochemistry Department.

Arthur presented several project possibilities to me as I began my fellowship with him in 1974. I chose to study the mechanism of the first purified cellular replicase, the DNA polymerase III holoenzyme (Pol III HE). The project turned out to be a deep dive into multiprotein complex purification.

Three important papers published from Arthur’s lab were used as the foundation for my work (8, 9, 10). An enzyme, termed Pol III∗, had been isolated, and it was concluded that it was a single subunit of 90 kDa. It was capable of replicating long single-stranded DNA together with a second activity, Copol III∗, that was attributed to a 77 kDa protein. Pol III∗ was distinguished from the earlier isolated Pol III by the latter being only able to fill short gaps in nuclease-activated DNA and a larger size evinced by gel filtration. The reaction on RNA-primed long single strands could be resolved into two stages. Upon incubation of Pol III∗, Copol III∗, ATP, and primed long single-stranded DNA, a complex, isolable by gel filtration, could replicate upon addition of dNTPs. Later, a Pol III HE was isolated that contained two subunits, identical to those previously identified as Pol III∗ and Copol III∗. It was concluded that Pol III was also 90 kDa, but the special properties of Pol III∗ somehow resulted from being a higher order polymer of 90 kDa subunits. This work was conducted by superb scientists and made multiple important contributions to our understanding of DNA replication. Nothing I write in the following is meant to be critical of the people who conducted the work. All findings described in this paragraph were confirmed, but the isolated enzymes were apparently quite impure, with the identified 90 kDa and 77 kDa components being contaminants. Purifying and identifying the authentic composition of the Pol III HE presented an enormous challenge.

I could resolve Pol III∗ and Copol III∗ activities, but 90 kDa and 77 kDa components did not emerge, even upon extensive purification. In parallel, I worked to purify Pol III HE, again being unable to reproduce previous work regarding subunit composition. Working hard, often monitoring columns into the early hours of the morning to overcome lability issues with speed, I was unsuccessful by the metric of reproducing previous work. Arthur became increasingly impatient. It was clear he thought I was not a competent scientist. Criticism went beyond what was appropriate, going to the personal level. Humiliation only strengthened my determination to solve the Pol III HE problem: I just put my head down and worked even harder, trying to ignore the toxic atmosphere in which I conducted my work. My good friend and fellow postdoc, Roger McMacken, provided important support as did the rest of Arthur’s group. After his arrival, Shlomo Eisenberg was also very supportive. He believed the quality of the intact Pol III HE that I provided allowed him to get an origin–dependent replicative system going.

When stressed, our unresolved personal issues reveal themselves and can synergize with our current life challenges, making matters more difficult. Arthur’s opinion of me resonated with my intrinsic sense of inadequacy, making matters worse for me. I lacked experience with how to successfully deal with conflict, and I became withdrawn, not feeling worthy to fully engage with a department full of outstanding developing graduate students and postdocs. In retrospect, I realize I should have drawn confidence from my previous successes and consulted more with my colleagues for constructive help with my challenging situation. My response at the time was simply to work harder, albeit largely in isolation. Successive 70–80-h weeks were stultifying. One habit helped: after an experiment ‘failed’, I stayed at my desk and mapped out the next approach, no matter what the hour. It was only by being clear on the next task and rekindling hope that I was able to wake up the next morning and enthusiastically reengage.

Eventually, I had purified Pol III∗ sufficiently that it was apparent that no 90 kDa component was present. A band at 140 kDa was prominent and its intensity paralleled the level of activity during chromatography. Upon showing these results to Arthur, he ordered me to change *Eco* strains used for the purification. I was deeply frustrated and discouraged, but complied.

I decided to focus on a strategy to purify, first, the intact Pol III HE. The approach was to purify the native enzyme present in low amounts using the enzyme’s intrinsic activity to guide purification. I came to know this enzyme as labile, sensitive to both low salt and dilution. I devised a purification procedure that avoided these liabilities. Cation exchange chromatography was a powerful step for purification of Pol III∗, like many polymerases. However, this step split the enzyme, resolving Copol III∗ from Pol III∗. By trial and error, I found that adding DMSO to the enzyme allowed binding of Pol III HE intact to a carboxy-based resin in high salt and that Pol III HE eluted as a single peak by keeping salt high and running a decreasing DMSO gradient. Hydrophobic interaction resins were not commercially available in the mid-1970s; however, the concept had recently been published using valyl-Sepharose. I reproduced the published procedure synthesizing valyl-Sepharose and found it a useful chromatographic step. An additional step yielded an enzyme, purified over 7000-fold, in which six components were detectable using low resolution SDS gels that cosedimented as an 11 S complex in a glycerol gradient. Pol III HE was resolved by phosphocellulose chromatography into two components with activities ascribed earlier to Pol III∗ and Copol III∗—they had to be combined to reconstitute Pol III HE activity. The Copol III∗ that I purified to near homogeneity was a single component of roughly 40 kDa. Pol III∗ had all the subunits found in Pol III HE except the 40 kDa band. The largest component in Pol III∗ was 140 kDa, as observed in my earlier purification attempts. While this work was ongoing, Charles Richardson’s lab brought Pol III to a high state of purity and it also contained a 140 kDa protein. Thus, I assigned the largest subunit in Pol III∗ as α and the 40 kDa protein as the β subunit of Pol III HE (11).

To gain further insight into what distinguished the simple gap-filling activity of Pol III from the replicase activity of Pol III∗, I developed a method to resolve a factor that, when added back to Pol III, reconstituted Pol III∗. I incubated Pol III∗ in high concentrations of *o*-phenanthroline, a Zn^++^ chelator, and mildly heated the mixture to avoid reformation of Pol III∗. This factor was purified to high specific activity and contained (within the limits of crude single-percentage SDS gels used at that time) two components that were found in Pol III HE that I assigned as γ (52 kDa) and δ (32 kDa). This factor, which I named the γ–δ complex (later called γ-complex or DnaX complex), was required to reconstitute Pol III HE activity in the presence of Pol III and purified β.

My postdoctoral work was funded by a fellowship from the Cystic Fibrosis Foundation that lasted 2 years. Arthur was unconvinced by my results and asked me to find another position before my fellowship ended. So, in 1975, at the beginning of my second year of postdoctoral training, I applied for faculty positions. I only learned in later years that Arthur’s recommendation said nothing about my contributions other than that both he and I were dissatisfied with my progress. That, plus the convention of my PhD advisor of listing himself as first author on all papers that came from his lab, for which he performed no experiments, put me in a very awkward place—an unfavorable recommendation from Arthur and no first-author papers. I sent off a very large number of applications and only received one interview—at the newly formed University of Texas Medical School in Houston. Jack DeMoss, an accomplished enzymologist who moved there from the University of California, San Diego, had taken the chairmanship of the department. I will remain forever grateful to him for giving me an opportunity. By a most fortunate coincidence, when I visited, Robb Moses, who was on the same multi-institutional campus in Houston, had invited Bob Lehman, from the Stanford Biochemistry Department, to give a seminar at the Baylor School of Medicine. Bob Lehman and Jack DeMoss met, allowing Jack to inquire as to whether the work I presented in my job interview seminar was all mine, because the results appeared not to support Arthur’s negative letter. Bob had taken the care to follow my work and vouched for me.

I was offered a faculty position, which I eagerly accepted. Despite the challenges, in 1976, I was an assistant professor at age 28, 6 years after my undergraduate degree. I am grateful for the synchronous events that gave me an academic pathway.

After I departed Stanford, Ralph Meyer, a professor at the University of Cincinnati, came to Arthur’s lab on sabbatical. He reproduced my purifications, finally giving Arthur the confidence to allow me to publish my findings, which provided an important contribution to future work in Arthur’s lab that required a pure, concentrated, cellular replicase and provided a foundation for my career.

To any graduate student or postdoc reading this who find themselves in a similar situation, I suggest discussing your issues with a supportive faculty co-mentor, asking advice on how to best approach your advisor. I further suggest interacting broadly with the labs within your environment and sharing your challenges with others. I also suggest balancing work with your personal life, getting adequate rest, and finding physical and/or meditative ways to minimize stress. Importantly, I also recommend seeking professional psychological support, realizing your potential, and not personalizing your struggle. Realize that any shame or inadequacy you feel are likely not real, just the product of your own thoughts, perceptions, and background.

## The start of an independent career in Houston

### Further defining the components of the first purified cellular replicase

Once in Houston, I decided to purify the simple-gap filling Pol III core activity to homogeneity to provide a reagent to support Pol III∗ and Pol III HE reconstitution studies. I hired an inexperienced but fast-learning and dedicated technical assistant, Weldon Crow. Weldon and I worked jointly in the purification of this activity. It required multiple steps and a 28,000-fold overall purification, consistent with its very low copy number in the cell. Terry Landers, another new faculty member in our department, introduced us to gradient SDS gels. To a young scientist, this will seem odd given how far science has progressed. SDS gels were just invented as I started graduate school, and the progression of tube gels to slab gels, then hand-poured gradient gels took a few years to develop. Using these high-resolution gels, three subunits became apparent: the familiar α of 140 kDa and two new subunits that I named ε (25 kDa) and θ (10 kDa). All of the proteins migrated in equimolar ratio during chromatography and in two-dimensional gels (12). The ε subunit was later shown by Maurice Bessman to be the product of *mut*D and the major proofreading exonuclease of Pol III HE (13).

**Figure 1:**
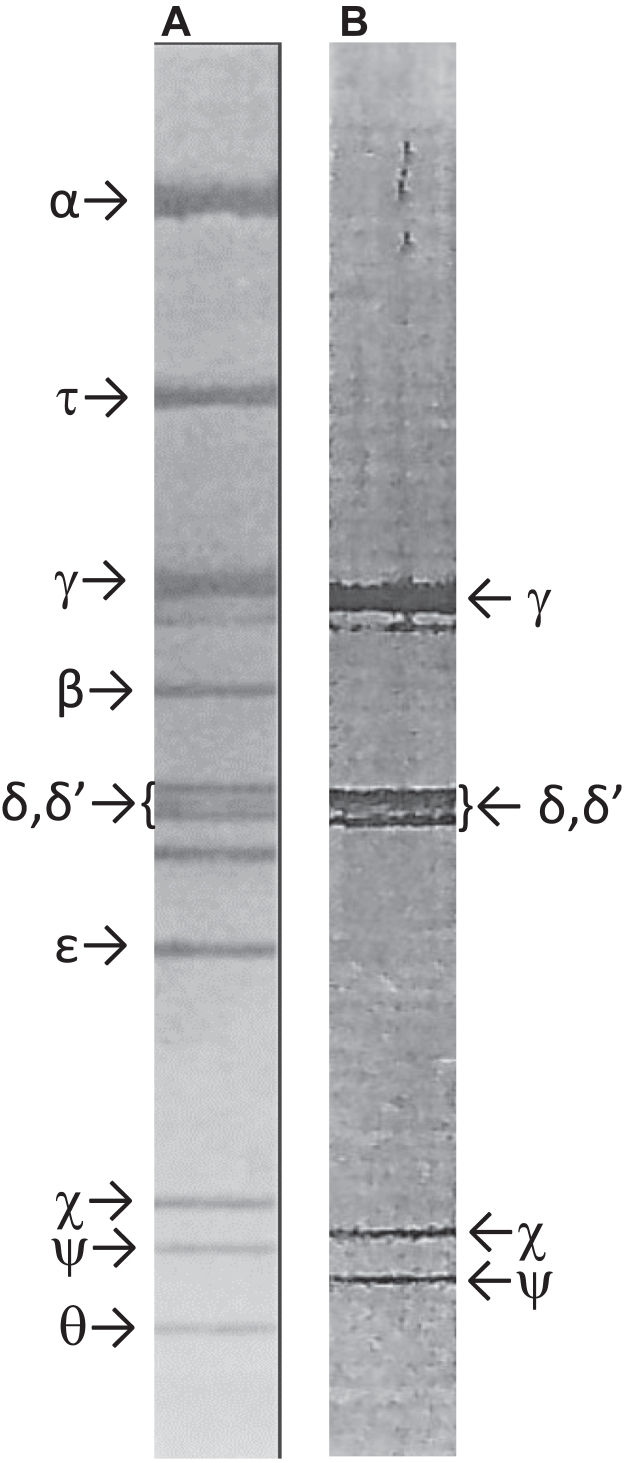
**Preparations of Pol III HE and DnaX (γ) complex purified at Stanford but run on higher resolution gels as described in text.***A*, DNA Polymerase III holoenzyme; *B*, γ complex.

Of note, using these new high-resolution gels with the Pol III HE I purified at Stanford, all of the presently known subunits of the Pol III HE were present and the enzyme was essentially homogeneous (Fig. 1 and Fig. 4 in (12)). Using gels similar to those used for the DnaX complex I purified at Stanford, all of the presently recognized subunits of the γ-complex (initially termed γ-δ complex) were present (Fig. 1; Fig. 1 in (14)). I had always looked at my postdoctoral performance as much less than it was. Primarily by reflecting while writing this article, I look back at my accomplishments as a postdoc as an enormous success: a 7000-fold purification of the first cellular replicase, Pol III HE, to homogeneity; purification of Pol III∗ and β; and discovery of the γ-complex that was responsible for conferring the special replicative properties upon Pol III.

### Discovery of Pol III’ and assigning the τ subunit of Pol III HE

In my early days in Houston, I explored the disassembly of Pol III HE to understand which subunits were associated with one another. A 15,000-fold purification of a form I called Pol III’ was a dimer of Pol III held together by a newly assigned subunit that I named τ (15). I discussed the notion that τ might hold the leading and lagging strand polymerases together at the replication fork, a notion that gained experimental support in later years. Bruce Albert’s lab had previously proposed interaction of the leading and lagging strand polymerases by an unknown, but functional link (16). Key to isolating Pol III’ was treatment with high salt. Fortunately, hydroxyapatite is blind to monovalent ion concentration, allowing resolution for the stripped away subunits.

### Processivity and β

I hired a highly competent enzymologist soon after coming to Houston. Kyung Johanson had excellent training in Perry Frey’s lab and moved to Houston because her husband had taken a position there—my good fortune! Kyung agreed to develop an improved purification for β and to study its role in the Pol III HE reaction. Kyung worked out a 10,000-fold purification of β from WT cells. She showed the protein to be a dimer of 37 kDa subunits and produced an antibody against β that inhibited the overall reaction. However, if a complex was formed on long single-stranded DNA in the presence of ATP, the subsequent elongation reaction was not inhibited (17), in agreement with earlier work with crude enzyme (8). Based on studies with the crude enzyme, it was proposed that β would dissociate after initiation complex formation, just like σ from the *Eco* RNA polymerase (Fig. 5.5 in (18)). However, in a clever and technically demanding set of follow-up experiments, Kyung showed that the β subunit was part of the elongation complex and that, presumably due to structural associations and/or rearrangements, became resistant to our polyclonal antibody (19). This showed that β was part of the elongation complex but did not yet show it was still functionally required. A key collaboration with Bob Bambara permitted that.

While I was at Stanford, Bob Bambara was a postdoc with Bob Lehman, establishing basic methods to address processivity, using Pol I. We agreed to address the processivity of the various forms of Pol III once we had established our own independent labs. We showed that Pol III alone exhibited low processivity (11 nucleotides); in contrast, Pol III HE was able to replicate an entire ∼6000 nt template without dissociation (20).

The key role of β was revealed fortuitously through an experimental error. Bob’s lab sent me data for an upcoming paper, and in it, I saw that much higher concentrations of β were being used than should have been required. Upon reviewing in detail, I noticed that ATP, hydrolysis of which is required to drive initiation complex formation, had been omitted. The template used for these experiments was poly dA, obviating the need for dATP, which can also drive initiation complex formation (21). This led to the demonstration that β could support processive replication by Pol III in the absence of any other auxiliary proteins (22), establishing β as the processivity factor for the replicative reaction. However, the properties of the Pol III + β complex were distinguishable from authentic Pol III HE by the lack of a stable isolable initiation complex. We proposed that one of the roles of ATP hydrolysis by the DnaX complex was to lock β in a state in which it did not equilibrate in solution (21). In extensions of this work, John Kuriyan and Mike O’Donnell showed an elegant ring-shaped dimeric structure for β that fit around DNA and could slide with the replication complex (23, 24), consistent with the term ‘sliding clamp’ for processivity factors first proposed by Bruce Alberts (25).

### Awards that facilitated my early career

Two awards greatly facilitated my early career. Early in my time in Houston, I obtained a career development award from the American Cancer Society. After being awarded tenure in 1981, I received a fellowship from the John Simon Guggenheim Foundation. I used it for a sabbatical at the University of Basel in Kasper Kirschner’s lab, learning to apply fluorescence energy transfer. I recall the kindness and support of Kasper and Jeff Schatz while being there. Being in Switzerland also strengthened my ties with Ueli Hübscher (who was a Kornberg lab postdoc whom I met when invited back for a seminar) and his wife Ursi. They were always generous hosts during trips to Zurich.

I recall, in the Houston phase of my career, respect for Jack DeMoss and Finn Wold, both senior colleagues who treated others with respect and had no agenda other than doing solid science and supporting the general good. I formed life-long friendships with Bill Dowhan and Henry Strobel while being there. I respected both for the way they did science but also for their emotional intelligence and the way they treated people. Another friendship with Robb Moses flourished *via* a variety of scientific meetings while we were both in the same field.

## Founding and directing a molecular biology program at the University of Colorado Medical School

The University of Colorado School of Medicine recognized that they had greatly fallen behind in modern molecular biology and formed a search committee to recruit a founding director of an interdepartmental molecular biology program. The notion was to help individual departments recruit faculty, but in some cases, faculty with complementary interests were not present. The molecular biology program provided a home. I was recruited to organize this program^1^. So, in 1985 at age 37, I found myself a full professor and founding director of an eventually very successful molecular biology program.

The form of the program was not predetermined. I felt strongly that the way to make the program a success was to focus on graduate training. Recruiting our own students greatly helped the individual labs and provided intellectual excitement and energy. Thus, we developed criteria for faculty membership in the program that included providing a suitable training environment. I resisted efforts from some clinicians who wanted access to our students to conduct molecular biology research in their labs but lacked the fundamental training to guide students. As one might expect, I received significant push-back on this latter point, particularly from chairs of some clinical departments.

The vitality of the program was enabled by bright faculty that had been recently recruited and who eagerly wanted to make their new academic home the best it could be. Peter Sarnow took over the graduate recruiting committee and attracted a superb group of students. David Pettijohn provided the idea of the program providing a mini-course annually on an exciting emerging topic and Alex Franzusoff headed up that effort. The mini-course was open to the entire community and was heavily attended. Arthur Gutierrez-Hartmann, who had great interpersonal skills, became the first student advisor and organizer of our first annual program retreat, which we held at the YMCA of the Rockies in Estes Park. Bob Sclafani took care of developing a curriculum and organizing team-taught courses. My only role was coordinating all, raising funds, and enjoying much of the work being done by these talented committee chairs!

Funding of the program came from the Medical School dean, supplemented by numerous grants. I am particularly appreciative of the support of Dean Joseph St. Geme, then Richard Krugman, and the critical help of Chancellor Bernard Nelson. I organized and served as principal investigator of numerous shared instrumentation grants that were all successful, providing critical instrumentation not only to the Molecular Biology members but the entire campus. Chancellor Nelson introduced me to Robert Glaser, who was then running the Lucille Markey Charitable Trust. We submitted a grant application and had close interactions through site visits, resulting in an award of seven million dollars which provided an enormous springboard for the success of the program. A private donor, Victor Bolie, contributed two million dollars to the program that we used as an endowment to support additional graduate trainees. Once the program was fully established, growing from nine to 44 faculty and having the requisite number of successful graduates, we obtained a training grant from the NIH. After that, I stepped aside in 2000 and the program successfully continued under the leadership of Dean Edwards, James DeGregori, and others to this day.

## DNA sequencing, gene arrangement, and cloning efforts

As I began my career, molecular cloning was in its infancy. Methods were crude, and initially gene sequencing was not available. While still in Houston, Mary Welch (26) obtained a *dna*E-containing restriction fragment from the Carbon Collection, one of the initial cloning libraries, that complemented *dna*E temperature-sensitive mutations. She was able to show *dna*E directed the synthesis of α. Once I moved to Denver, Sanger sequencing had been developed for use on long polyacrylamide gels. It took a year back then just to sequence a single gene. Henry Tomasiewicz sequenced the *dna*E gene and showed it to be on an operon with *lpx*A and B (27).

Ann Flower subcloned and sequenced the *dna*X gene and showed it to contain one ORF encoding both the γ and τ subunits of Pol III HE (28). The structural genes for these subunits had been initially mapped, genetically, as distinct genes, *dna*Z and *dna*X (29, 30). Proposals had been made that γ arose from the proteolysis of τ (31). Ann located a site representing the end of γ within the ORF that was translated into τ. We hypothesized that this occurred through translational frameshifting (32) and later followed up with experiments demonstrating that mechanism (33). Both Jim Walker’s and Arthur Kornberg’s labs independently arrived at the same conclusion, publishing a few months before us (34, 35).

Molecular clones and protein expression vectors were already available for the genetically characterized Pol III HE structural genes. With advancing technology, Jeff Carter and Mary Ann Franden embarked on cloning the remaining structural genes for subunits of the Pol III HE that had not been identified genetically (36, 37, 38, 39, 40). The same feat was accomplished concurrently in Mike O’Donnell’s lab (41, 42, 43). This provided the ability to overproduce the corresponding proteins, overcoming the limitations of purification of these low-abundance proteins from native *Eco*.

## Mapping Pol III HE subunit interactions at the domain level

One of the avenues we pursued using these expanded quantities of protein was to map the interactions of the subunits down to the domain level. Domains were determined initially by proteolysis, then functionally by making deletions in expression clones or directly from structures as they became available from the productive O’Donnell-Kuriyan collaboration. Our favored approach was to immobilize one subunit by the amino- and, separately, by its carboxyl-terminus and to survey domain interactions. By phage screening, researchers at Affymax had identified a 13-amino acid sequence that could be biotinylated by the endogenous *Eco* activity (44). Millard Cull worked at Affymax before joining our lab and alerted me to this important work. Graduate student Deok Ryong Kim pioneered development of our general methodology by tagging Pol III α subunit deletions of varying length either on the amino or carboxyl terminus with this biotinylation tag. They adhered to BIAcore chips modified in our lab to present covalently attached streptavidin. The streptavidin chips bound biotinylated α derivatives and, after washing, other subunits were passed over, and the kinetics of binding and release assessed to determine a K_d_. Streptavidin biosensor chips are now widely commercially available and the Affymax peptides broadly used. To my knowledge, we were the first to exploit this technology (45). Using this methodology, Deok Ryong Kim showed that τ bound to the carboxyl-terminal domain of α and that β bound to an internal domain near the carboxyl terminus (aa 542–991) of the 1160 residue α (46). Anna Wieczorek showed that the N-terminal PHP domain of α binds the ε proofreading subunit (47).

Graduate student Dexiang Gao used similar technology to show that domain IV of the five-domain τ binds the replicative fork helicase, DnaB. Exploiting the density of domain IV monomers on the BIAcore chip, she demonstrated that at least two DnaB monomers within the hexameric helicase were required for optimal interaction (48). She showed that the extreme C-terminal domain V of τ bound α (49) and that τ domain III, shared with γ, is solely responsible (energetically) for binding δ and δ′ and also χ-ψ (50).

Min-Sun Song pulled this full circle (literally) by showing domain III of the δ′ subunit binds τ and γ and supports cooperative DnaX complex assembly of the DnaX complex pentameric ring (51). Domain III of δ, homologous to domain III of δ′, γ and τ, binds δ’ (52). Brad Glover showed that domain III is responsible for formation of the oligomeric forms of γ and τ (53). Matthew Olson showed that χ-ψ, upon binding to DnaX, increased its affinity for δ and δ′ to a physiologically relevant range (54).

## A novel nuclease that is part of the proofreading apparatus of bacterial replicases

The PHP domain to which we showed ε binds is named after a putative domain with homology to phosphatases. The Koonin laboratory found these sequences in bacterial *dna*Es and named them PHP after polymerase and histidinol phosphatase (55). They proposed that they might function as pyrophosphatases. We pursued this potential activity initially using *Thermus thermophilus* (*Tth*), in part motivated by close homology and also being able to distinguish any activity identified from mesophilic contaminants by heat denaturation. Natalie Stano found that, instead of a pyrophosphatase, it functioned as a 3’->5′ proofreading-type exonuclease^2^ (56). She used highly purified *Tth* α. The *Tth* PHP exonuclease and polymerase activities decayed slowly and in parallel at 55 °C. The *Tth* PHP domain showed close homology to *Eco* YcdX. The structure of YcdX revealed a Zn^++^ trinuclear center. Treatment of *Tth* PHP with the Zn^++^ chelator o-phenanthroline destroyed activity.

In *Eco*, the PHP domain of Pol III α binds the ε proofreading subunit. *Bacillus subtilis* (*Bsu*) PolC contains its ε proofreading homolog within the same polypeptide as the polymerase. Interestingly, this *Bsu* ε-like sequence is inserted in the middle of the *Bsu* PHP (47). This suggests some sort of cooperation, such as a co-processing activity, with one being better adapted to a specific lesion than the other (56).

In unfinished work, Natalie searched to identify an *Eco* PHP activity analogous to the *Tth* PHP. She expressed and purified overproduced Pol III α in a strain in which θ was deleted, that allowed us to remove the low WT levels of ϵ by passing purified α over a θ affinity column (θ binds ε tightly and is the third protein in the Pol III α-ε-θ core). To provide an assay, Natalie surveyed differences in activities between *Tth* PHP and *Eco* ε, hoping to develop assays to distinguish ε-like from PHP-like activities. *Tth* PHP was more active in the removal of 3′-Pi-nucleotides while inactive on 3′ α-thiol-phosphate bound nucleotides. *Eco* ε was active on the latter, degrading one of two diastereomers. Natalie’s *Eco* α possessed nuclease activity even when highly purified, but we could not be certain that it was intrinsic to the α chain. To test this, we planned to add nuclease-free *Eco* τ, shifting the Pol III to a dimer, separable by gel filtration. If we found a corresponding shift in nuclease activity, that would have provided a conclusive result. She did not have the large quantities of α from the θ-deletion strain required for Pol III’ reconstitution when we ran out of funding. I hope someone will take up this project. I urge any prospective investigator not to be discouraged by the lack of complete conservation in the *Eco* PHP domain. Even the oxygen of peptide amides serves as a Zn^++^ ligand in some cases. We recognize, for *Eco* PHP, addition of an inactive ε mutated in an essential acidic active site residue might also be required for conformational or structural reasons.

## The *Eco* replicase is an asymmetric dimer

Kyung Johanson showed that ATPγS supports initiation complex formation of the replicase on DNA but only to 50% of the level achieved with ATP (57). Curiously, the addition of ATPγS to complexes formed in the presence of ATP led to rapid dissociation or inactivation of one-half of the initiation complexes. With our knowledge that a τ dimer held two Pol III assemblies in a complex (15), we proposed that the two halves of the replicative helicase were functionally asymmetric. We hypothesized that this may be a reflection of an asymmetry of the replicase with distinguishable leading and lagging strand halves (58).

Brad Glover extended this work to show that the two-fold difference in initiation complex formation in the presence of ATP relative to ATPγS was due to half the amount of initiation complex formation in the presence of ATPγS (59). Using primed circles as templates, Brad demonstrated that replication of a second strand of DNA starting with initiation complex formed in the presence of ATPγS required ATP.

Starting with initiation complexes formed in the presence of ATPγS by a biotinylated primer bound to avidin beads, then washed, a second molecule with a nonbiotinylated primer could be bound and replicated if ATP was added. Formation of a dimeric replicative complex by this two-step procedure allowed Brad to show that the asymmetry of the replicase was retained upon formation of a dimeric complex. Tests with reconstituted rolling circle replication forks showed that the lagging strand half of the replicase was inactivated by ATPγS, supporting the asymmetric dimer hypothesis.

### Structural basis

At one level, a dimeric replicative complex with a DnaX core with a circular pentamer containing two τs and one each of γ, δ, and δ′ bound to single subunits of χ and ψ is obviously asymmetric. Brad Glover further explored the nature of this asymmetry using protein cross-linking with natural holoenzyme isolated from WT *Eco* (60). He found a cross-link between the γ subunit and ψ and δ’. This indicates that γ occupies a unique position within the DnaX complex, distinct from τ-positioning.

## Personal crisis-induced evolution

Earlier, I described some of the difficulties I encountered during my postdoctoral work at Stanford as both external and internal. I felt an intrinsic sense of shame and not being good enough. Once independent, these insecurities drove me to be very demanding of myself and those in my lab, and this stressed my relationships. Imprinting from previous mentors synergized, and my inability to deal effectively with conflict and my introverted withdrawn nature isolated me. I was viewed as a ‘hard ass’ mentor and deservedly so. I expected everyone to have the highest level of dedication and perseverance, working toward the best faculty position they could find. I had great relationships with the brightest and hardest-driven lab personnel. I worked with them with only general guidance and helped with decisions only as needed. I had developed confidence in graduate school that way and I expected everyone else to as well. Problems arose when students needed more guidance than I gave. In retrospect, I realize I thwarted the success of some students and postdocs by not providing enough guidance and supervision.

I rarely celebrated success or congratulated lab personnel. There was always the next problem and that was my focus. This approach took an emotional toll on both me and on some of the people in my lab.

This and the accompanying end of an important relationship led to a period of significant depression in the early 1990s. During the worst of it, I often worked door-closed in my office and minimized human contact to what was necessary to keep my lab and the molecular biology program running. Outside of my office, I kept my ‘game face’ on and, I suspect, few realized the struggles I was going through. Unfortunately, in our field, there is a code of “work over any adversity.” This leads to scientists failing to seek professional help until things get really bad. Fortunately, I recognized I needed help.

I had the good fortune of finding a very talented psychiatrist who had keen insight and allowed me to feel understood. This provided the professional support and guidance I needed. With years of therapy, the layers fell away, and I no longer felt the initial strong drive in my career that was based on the intrinsic feeling of not being good enough. Indeed, I had an epiphany, replacing the initial driver with a sense of gratitude for working in my chosen field. I always had a love of science and I greatly enjoyed learning how the replication apparatus works. Focus was stimulated by reading a line in a poem by Marilyn Nelson, *Faster Than Light*: “If I had to remain anonymous, would I publish? Would I write poems at all?” I reflected on this and realized the answer early in my career was likely no, but at the stage I found myself, a resounding yes! Some of us might take self-importance from current positions and accomplishments, but nearly all of us will become anonymous after our careers end and science progresses. This renewed my desire to pursue research for the foundational knowledge that it provides to humankind. Everything we do likely impacts the future accomplishments of others in unfathomable ways. This made me even more grateful to be doing my work. I feel like a much different person now.

Scientists are not given training in supervising lab personnel nor is there much impetus for increasing our emotional intelligence in early scientific training. I urge junior faculty to evaluate these ‘soft’ skills (or ask others with whom they interact!) and to seek appropriate training in these skills and to get help, if indicated, as soon as possible. It might initially seem to be pulling you away from your all-consuming research, but you will find that it ultimately makes you more effective and helps you enjoy your personal and professional life^3^.

## FRET, cross-linking to study subunit rearrangements during initiation complex formation

Mark Griep exploited the FRET program I began as an outgrowth of my sabbatical with Kasper Kirschner to study subunit dynamics in β (61) (that was erroneously initially characterized as subunit dissociation, but we now know from equilibrium sedimentation studies of Garry Dallmann in my lab that this was just the opening of one of the two dimer interfaces in circular β). Perhaps future studies will show that this open form of β_2_ is trapped by the ATP-liganded form of DNA complex, leading to clamp loading. We also used FRET (along with cross-linking studies of graduate student JoAnna Reems) to show that β is positioned 22 nt behind Pol III∗ in the elongating complex (62, 63, 64). JoAnna showed that α contacts the first 13 nucleotides of the primed template and that γ of the DnaX complex contacts initiation complexes 18 nucleotides behind the primer-template.

## Assembly of the DNA polymerase III holoenzyme

A particularly thorny issue in understanding assembly of the replicative polymerase is how the two *dna*X gene products–τ and γ–are assembled into the same complex. DnaX is remarkable in that it forms complexes with distinct stoichiometries, depending on its bound partners. Garry Dallmann showed that both γ and τ exist as tetramers in solution by themselves (65). τ, when bound to Pol III, is a dimer, forming Pol III’ (15). Mixing either a γ or τ tetramer with δ, δ′, χ, and ψ results in a trimeric DnaX assembly (66). A monomeric form of γ exists in an equilibrium of γ _4_ (65).

Simple mixing of purified individual subunits results in DnaX complexes containing exclusively γ or τ (66, 67, 68). In contrast, γ and τ co-assemble when produced *in vivo* from a five-gene artificial operon from an expression plasmid (66). Art Pritchard developed an *in vitro* assembly assay using biotinylated τ that could be pulled from solution with streptavidin beads, washed, and its binding partners recognized (68). γ was found to assemble into τ-containing complexes in a time-dependent manner, using the remaining complement of subunits to drive formation of a stable complex and arresting DnaX subunit exchange. The assembly reaction is stimulated by Pol III, potentially important because Pol III interacts with τ, forcing dimer formation. γ does not bind Pol III. δ and δ′ do not stably bind to a τ dimer. We hypothesized that in cells, τ may be sequestered by the α subunit of Pol III soon after synthesis. This would provide a situation for free γ (in equilibrium with γ_4_) to assemble into a labile trimeric mixed DnaX assembly that could be trapped by binding a δ, δ′, and χ-ψ. A potential scheme and supporting rate and equilibrium constants is presented elsewhere (69). This problem remained a focus of mine until my lab closed. If time and resources had permitted, I would have directed experiments starting with Pol III’, mixing with dilute γ (to favor monomer formation), and then diffusing in the remaining subunits to find conditions for assembling γ stoichiometrically into a unique location like in natural Pol III HE.

## Replication in gram-positive bacteria

Gram-positive bacteria have two DNA Pol IIIs with PolC and DnaE as the catalytic subunits. To explore their respective functions, we developed expression vectors for the known or predicted *Bsu* replication proteins, purified, and used them to reconstitute a complete rolling circle replication system dependent on 13 proteins (70, 71). Glenn Sanders found that PolC is the only Pol III needed to replicate the leading strand but that both PolC and DnaE are required for lagging strand replication. We found that under physiological conditions that PolC cannot use an RNA primer but DnaE can. After initiation of elongation, DnaE is replaced by PolC, which performs the bulk of lagging strand synthesis. John Zinder followed up and showed that DnaE in the presence of the *Bsu* clamp loader (τ-δ-δ′) and β clamp is highly processive and is actively evicted by PolC (72). Thus, on the lagging strand, DnaE performs the same function as Pol α in eukaryotic systems before handoff to Pol δ. This remained a keen area of interest until the lab closed. I believe this will provide a good system to determine mechanisms of polymerase exchange, a process important for eukaryotic lagging strand replication and the exchange of error-prone polymerases with replicases in all organisms.

During my final sabbatical, we collaborated with Silvia Ayora’s lab in Madrid to reconstitute the replication of subtilis phage SPP1 (73). The system was dependent on *Bsu* elongation apparatus (DnaE, PolC, clamp loader, clamp) plus primase. *Bsu* single-stranded DNA binding protein (SSB) could support SPP1 replication, but it would not support PolC elongation, providing a mechanism for the phage to subvert the cellular replication system. We found that the SPP1 origin-binding protein also could mimic *Eco* PriA in initiating replication on non-origin containing flapped DNA substrates. Four phage-encoded proteins were required: helicase, helicase loader, origin binding protein, and SSB.

In addition to the scientific progress made during my sabbatical visit, important personal interactions resulted. I greatly appreciate the friendship and support of Silvia Ayora, Juan Alonso, Carmen San Martin, Jose Maria Carazo, their families, and laboratories.

## Chaperoning of Pol III onto newly loaded β during initiation complex formation

Chris Downey demonstrated that τ-containing DnaX complexes chaperoned associated Pol III onto newly loaded β (74). The presence of only one τ in DnaX complexes is sufficient to support the full rate of initiation complex formation—100-fold faster than complexes that contain only γ, bringing the overall rate to 25 to 50/s, compatible with the rate required to support ongoing Okazaki fragment synthesis at the replication fork. Analyzing DnaX complexes containing one or more K51E variants revealed that only one ATP-binding site within the trimeric DnaX is required to support initiation complex formation, but the full complement increases the rate about 1000-fold. Chris found that in ATPγS-supported initiation complex formation, the nucleotide analog is hydrolyzed at a rate equivalent to the rate of initiation complex formation at about 1/1000th the rate of the ATP-supported reaction.

## Move to the University of Colorado at Boulder

For the last decade of my career, I moved to the University of Colorado at Boulder in 2006, my first faculty position in an Arts & Sciences environment. The move was motivated by the opportunity for a change of environment. I initially greatly enjoyed the breadth—attending seminars ranging from organic chemistry to physics—and the diverse group of faculty with whom I associated. I had spent 21 years at the University of Colorado Health Sciences Center (as it was called then), had made multiple structural contributions to the school, and was feeling stale. If I had spent most of my career at Boulder, a move to the University of Colorado Medical School would have been just as attractive—both are strong institutions. I had great interactions with faculty there and enjoyed the support of the administration.

## Circling back: extra research required to test our previous work

There were two occasions during my career in which I felt we had reached firm conclusions regarding the composition of the Pol III HE and its interactions, but these later received challenges from multiple other studies. Faced with such a situation, does one trust the true answer will eventually evolve or should one turn back, devising unambiguous approaches to test the original claims? We pursued the latter route, but at the expense of advancing other projects.

### The β interaction site within Pol III

Using a series of deletion variants of the Pol III α subunit, we showed that a domain roughly between residues 542 and 991 bound the β sliding clamp processivity factor with high affinity (46). Deletion of 48 residues from the carboxyl terminus of Pol III α abolished τ binding with only a modest effect upon β binding (45). Subsequently, Nick Dixon and colleagues using bioinformatics and yeast two-hybrid analysis determined a universally conserved sequence that is most critical for β binding at residues 920 to 924 (75). Thus, by two independent labs coming to congruent conclusions by two very different approaches, we had high confidence in our conclusions.

Then, a claim was made that the β-binding site was located in the C-terminal 20 residues of α (76), in disagreement with our earlier results. This work concluded that an interplay of τ and β with the Pol III α C-terminus, modulated by DNA, provided the basis for Pol III cycling during Okazaki fragment synthesis (77, 78).

To address this controversy, we took a three-pronged approach—a genetic test of the importance of β-binding sequences, functional assays of the mutants generated, and a direct physical-binding analysis (79). Mutation of the internal site we initially identified eliminated β binding in the context of full-length α and abolished the ability of the resulting α to participate in replication *in vivo* or *in vitro*. In contrast, mutations of the C-terminal residues of α exhibited near WT phenotypes *in vivo* and could bind β and participate in processive DNA synthesis *in vitro*. Subsequent structural work reinforced our conclusions (80). Thus, our initial conclusions were valid.

### The composition of the DnaX complex within the Pol III HE

Initially, the composition of the DnaX components arose from finding that DnaX tetramers were converted to trimers upon binding the remaining DnaX complex subunits (66). Based on that finding, we proposed that native DnaX complex contained one copy of γ and two of τ to accommodate the known τ-requirement for dimerizing Pol III within the replicase. A preponderance of evidence for the presence of one copy of γ within Pol III HE arose. All preparations of native Pol III HE contain both τ and γ. Jim Hawker showed that immunoprecipitation using a monoclonal antibody directed against the unique C terminus of τ coprecipitated γ, showing it was part of the same complex (81). Protein-protein cross-linking conducted by Brad Glover demonstrated that γ occupied a unique position relative to the other components of the DnaX complex (60).

These findings were followed by some confounding developments. One lab reconstituted DnaX complex using only the τ subunit in the absence of γ and, as would be expected, showed it bound to three Pol IIIs, consistent with the known tight interaction of τ with Pol III α (82). It was proposed this might be the form of Pol III HE active in cells. Then, a British group, using a microscopic approach with fluorescently tagged proteins within cells, came to the same conclusion (83). The data within this paper did not support this conclusion: results showed 3.1 ± 1.1 α subunits and 3.1 ± 0.8 τs per assembly. Using a *dna*X mutant that only expressed τ increased the stoichiometry of τ at the replication fork by one-third (83), an observation consistent with γ being present in authentic Pol III HE.

The field was receptive of these new findings, but we felt the preponderance of evidence weighed against them. Paul Dohrmann and I devised a strategy to test unambiguously whether authentic γ was assembled into Pol III HE and demonstrate that the γ-like band we observed was not an artifact arising from processing of τ into a shorter protein (84). We obtained a version of *dna*X that was mutated in the frameshifting site so that it only produces full-length τ, generously provided by David Sherratt. A version of γ that contained a biotin tag on its C terminus was expressed from a low copy vector that we showed produced γ at about the same level as from chromosomal *dna*X. We purified Pol III∗ and showed it contained approximately one molecule of biotinylated γ per Pol III∗ assembly, putting this issue to rest. In collaboration with Susan Rosenberg’s lab, we found that mutants that express only τ in the absence of γ show increased sensitivity to UV light and a reduction in DNA Pol IV–mediated mutagenesis associated with double-strand break repair and impaired maintenance of an F′ episome (84).

This deep dive into proving that γ was an intrinsic part of the *Eco* replicase came at a cost. We proposed the experiments described above to test the presence of γ in Pol III HE as one aim of our final unsuccessful NIH proposal. Other aims proposed the elegant studies by Quan Yuan (85) (next section), the chaperoning of Pol III onto newly loaded β performed by Chris Downey (74, 86), a DNA Pol IV study performed with Mark Sutton (87), and our genetics-based approach finding sites within *dna*E required for forming initiation complexes *in vivo* (88). One reviewer of this unsuccessful application stated that the field had moved on and McHenry was still worried about whether γ was a component of the Pol III HE. This, in my view, was an unfair assessment. One lesson: reviews are by people, including myself, who have favored approaches, prejudices, and are fallible. The peer-review process is not always fair. Any principal investigator should keep this in mind and always have a backup strategy.

## Communication between replicase and primosome at the replication fork

Studies of special interactions at the replication fork provided a long-time interest, both in Denver and Boulder. These studies were highly dependent on cooperation with Ken Marians and started with a survey of the activities required for the synthesis of reconstituted rolling circle reactions using flapped DNA substrates (89, 90). Among other things, we found that lagging strand synthesis, after initiation, was not sensitive to dilution of Pol III, suggesting it was being held in place by protein–protein interactions. An interaction with dimeric τ to hold the leading and lagging strand polymerases together provided one possible mechanism.

**Figure 2:**
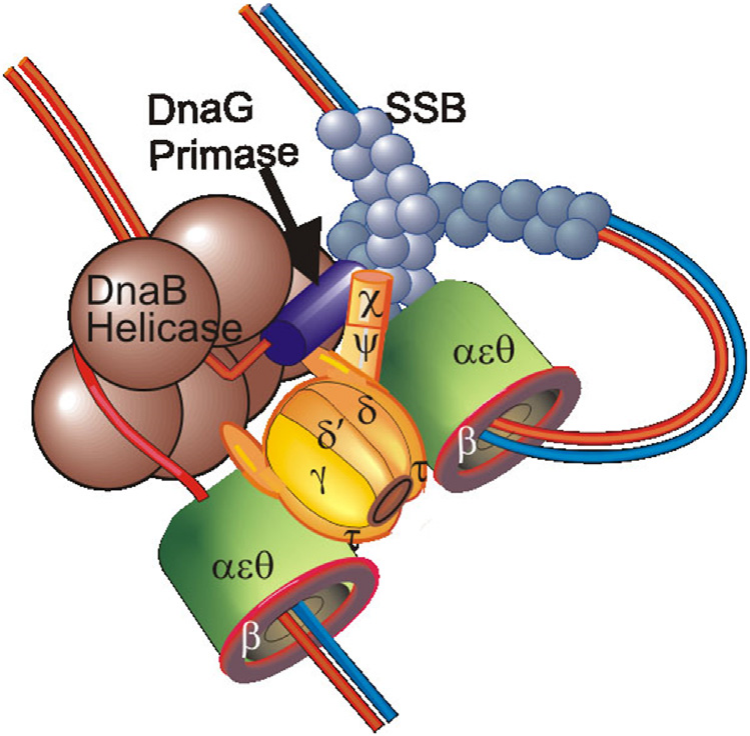
**Diagram of protein interactions at the replication fork**.

Using rolling circle templates, it was found that reactions with added τ progressed 10-fold faster than with the γ-only DnaX complex (91). We detected an interaction of τ with DnaB, consistent with a τ tether binding the DnaB helicase and Pol III directly stimulating the rate of helicase progression. Thus, the elongation apparatus and the primosome are physically and functionally coupled. As described above, Dexiang Gao also demonstrated a high affinity interaction of DnaB with τ that required binding of at least two DnaB protomers (48). This suggests that the two τs within the elongation process contact helicase (Fig. 2).

When Ken Marians’ attention turned to other important interests, he generously set us up with all his primosomal protein expression vectors, purification protocols, and helpful advice. Using these proteins, Carol Manhart showed that PriA serves as a checkpoint by blocking replicase advancement on replication forks that lack DnaB helicase (92). In the absence of PriA, dimeric Pol III elongating only the leading strand can slowly open helices, dependent on a τ-ψ-χ-SSB link to the lagging displaced strand (93) 4. Using synthetic model replication forks that contained phenyl diazirine linked to the 5-position of uracil at unique positions, Carol Manhart showed that PriA interacts with both sides of a fork. Addition of PriB and DnaT did not displace PriA. Addition of DnaB helicase and DnaC helicase loader led to PriA release, replaced by helicase contacting the duplex region on the lagging strand template and contacts with the displaced single-strand on the leading half side. We pursued this study for its own right but with an eye toward informing us about the binding positions and assembly mechanism of the *Bsu* primosome. *Bsu* uses a novel set of proteins that, unlike *Eco*, function in both replication restart and origin-dependent initiation. Alas, the lab closed before we could initiate those studies with the system Carol so arduously set up.

A related issue of interest was the mechanism used to recycle the lagging strand replicase to the next primer synthesized at the replication fork. Two hypotheses were put forward prior to our work. In one, the collision model, the elongating polymerase encountering the 5′-end of the preceding Okazaki fragment triggered cycling (77). In the signaling model, the synthesis of the next primer at the replication fork was somehow communicated to the lagging strand replicase, causing dissociation and migration to elongate the new primer (90). Paul Dohrman showed that at a 10-nt gap, the replicases dissociated with a half-life of 15 min. Complete filling of the gap only increased the rate of dissociation 7.5-fold to t_1/2_= 2 min, far too slow to support the ∼0.1 s cycling to the next primer at a replication fork (94). This increase in release after gap filling could be important in mismatch repair and other repair processes in which Pol III participates. Cross-linking showed that only α contacted the 5′-end of the preceding model Okazaki fragment, disproving models in which τ was the sensor, at least in the context of the collision model for which it was proposed.

Quan Yuan did the heavy lifting, testing the signaling model using flapped rolling circle templates with a very asymmetric nucleotide composition between strands (initially developed for our *Bsu* studies). We took a lead from Charles Richardson’s lab who showed that use of minicircle templates permitted concentrations high enough that polymerase and helicase could be made limiting, avoiding potential artifacts from these activities acting on initial products (95). We also took technical guidance from Steve Benkovic’s lab who showed the rates of elongation could be modulated on templates with asymmetric nucleotide composition by modulating the concentration of the relevant dNTP (96). In our case, we instead used nucleotide analogs (dNDPNP) that had a lower affinity for Pol III and a slower intrinsic rate of incorporation. The 409-nt flapped rolling circle template used had a 50:1 asymmetry in G:C distribution, with G being present predominantly in the lagging strand product. This allowed us to ‘dial-in’ the rate of elongation by varying the relevant dNDPNP in the presence of the remaining three dNTPs. We could also selectively block elongation of Okazaki fragments by varying the ddGTP:dGTP ratio. Quan used this system to demonstrate that Okazaki fragments could be terminated by ddGTP incorporation, leaving gaps between fragments, but otherwise the level of Okazaki fragment synthesis continued unimpeded, consistent with the signaling model and inconsistent with the collision model because Okazaki fragment completion would be required for cycling. Ken Marians had shown earlier that exogenous primers could be added to bypass the need for primase (97). Quan showed that by adding exogenous primers, the same result was obtained upon addition of ddGTP, suggesting that it was the presence of a new primer, not the synthesis of a primer *per se*, that was the signal for cycling. Similarly, the rate of Okazaki fragment synthesis could be slowed 20-fold with dGDPNP. These Okazaki fragments also had gaps between them and lagging strand synthesis continued unimpeded.

### Polymerase exchange into the replication fork

Using the rolling circle replication system developed by Quan, we examined polymerase exchange into the replication fork. Although addition of a dominant-negative D403E Pol III α into reaction premixes inhibited replication in proportion to the D403E α added, its addition after establishing ongoing duplex replication had no effect. Only when D403E was added in complex with τ-containing DnaX complex was it able to inhibit ongoing replication (87). One explanation for this is that the challenging Pol III∗ needs to interact with an unoccupied DnaB protomer within the hexameric helicase through the known τ−DnaB interaction to permit exchange. To our surprise, in a survey of DnaX complexes containing one, two, or three τs, only one τ is required for rolling circle replication. We proposed that redundant interactions of τ with DnaB may stabilize the duplex fork sufficiently under the reaction conditions used.

In contrast, in collaboration with Mark Sutton, we found that Pol IV can exchange into replication forks by itself, distinguishing the exchange mechanism of this error-prone DNA polymerase from the natural replicase (87).

## The final challenge: a crisis induced by a funding lapse

I was always aggressive at fundraising, both for the programs I participated in and for my own research. The lab worked with a huge array of different proteins, multiplied by a large number of mutants, variants, and truncations. This required a large professional staff ranging from a 250 L fermentor operator to employees that maintained a system for tracking and storing our reagents, expression vectors, and monoclonal antibodies. We had an expansive array of equipment, requiring large maintenance contracts. In other words, our lab operation was not readily down-scalable. Times changed during my scientific career. NIH review committees were reconfigured such that, for my area of research, alternative committees no longer existed. Great progress was being made in other areas of science in eukaryotic organisms, etc. Impact became a major criterion for grant merit review—and mature fields such as mine were often judged less impactful. I am not saying I did not see an end eventually coming, just not so soon. When I went to Boulder, I had six grants. Ten years later, I ran into a period with reviews that did not fall within the payline, all from the same review panel. For the first time in nearly 40 years, I was not funded.

I had three graduate students in my lab. I knew the department would support them until their graduation but with a sharp eye toward costs and time for the rigor I always required. My professional staff would have to be laid off immediately. I knew my graduate students would not prosper. So, I negotiated a painful but necessary deal. The Arts & Sciences Dean’s office agreed to a phased retirement arrangement that decreased my salary, and those salary savings were paid in one lump sum to my lab, allowing me to maintain two of my professional staff and operate at a functional level until my students graduated. I agreed to retire no later than the end of the 5-year period. I was able to secure funding within the next few months from the National Science Foundation. Those funds and my own salary plowed back into the lab allowed for the orderly completion of some important unfinished projects and their successful publication, including a couple of the best papers that ever came out of my lab. Nevertheless, the fate of the lab was sealed. One of my support staff, Paul Dohrman, PhD, stayed on and helped wrap up critical final papers. I am deeply grateful for his loyalty and selflessness in a tense high-pressure situation. If he had left to pursue another opportunity, several of our final important papers would not have reached publication, and the dissertations of my final students would have been compromised. Paul also took care of the tedious and difficult task of giving away thousands of vials of reagents to seven different labs in four countries and allowed us the satisfaction of a strong finish.

**Figure 3:**
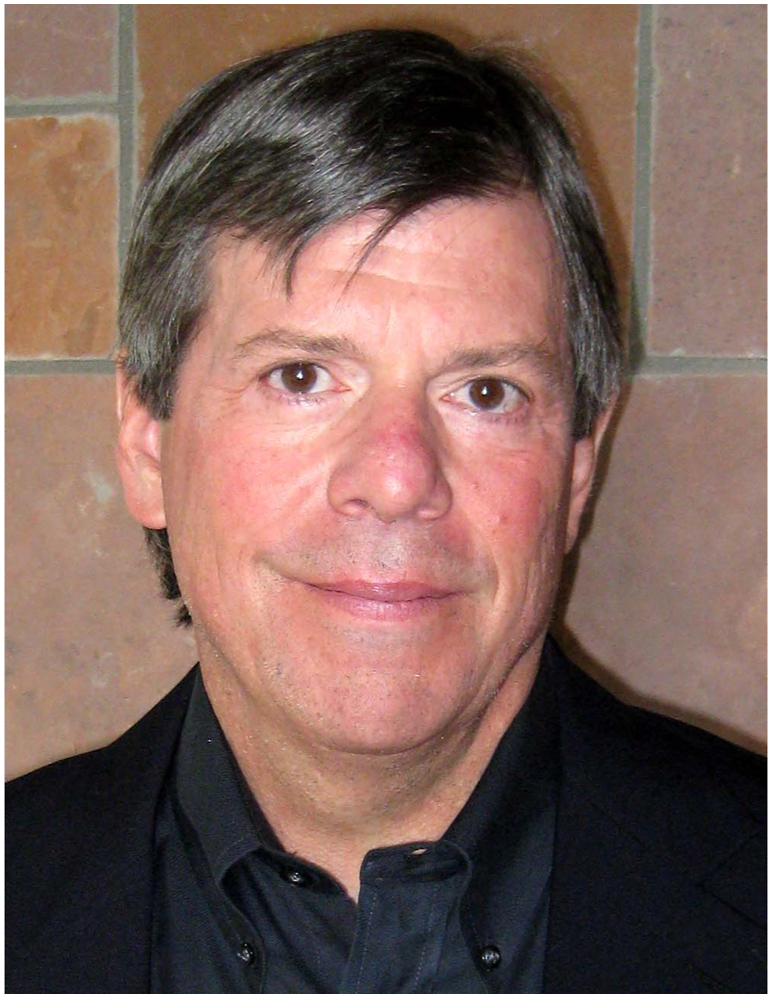
**Photograph of author in Boulder on or before 2013**.

We knew months in advance from funding projections when the lab would close. It was a quiet ending. After a celebratory dinner a few nights before, I thanked Paul one last time for his help and support. On the final day, I completed the packing in my office, staying until everyone was gone. I went into the lab, quietly walking around, reflecting, and touching equipment that I had used when I was personally more involved in hands-on research—a very poignant moment. I turned the lights off, locked the door, and took a flight the next morning to San Francisco, where I now live an active and interesting life during my retirement. I do not regret my decision about how the lab ended. I still think it was the best path through a difficult situation. But I do miss the focused meditative thinking that goes into figuring out how chosen molecular aspects of life function while pursuing the next important question Figure 3.

## Footnotes

1 When I discussed wanting to take the Colorado position with my chair, Jack DeMoss, he listened with a slight smile and calm demeanor. He told me it sounded like a prestigious position. He wanted the best for his faculty and was fully supportive. He even gave me permission to go on my already-arranged sabbatical in Switzerland

2 A hint of this activity came from Jim Bullard whom I supervised at Replidyne. He showed that Tth Pol III HE could extend a primer with a mismatched 3′-end.

3 My intention with taking such a deeply personal dive in this article is to provide a helpful case study for trainees struggling with their advisors and for faculty inappropriately dealing with their students and postdocs

4 These observations were confirmed and extended by Nick Dixon’s lab. In addition to the interactions we found to be necessary for Pol III HE strand-displacement, a previously unknown ε- β interaction was revealed (98).

## References

[1] Santi D.V., McHenry C.S. (1972). 5-Fluoro-2'-deoxyuridylate: covalent complex with thymidylate synthetase. Proc. Natl. Acad. Sci. U. S. A..

[2] Santi D.V., McHenry C.S., Perriard E.R. (1974). A filter assay for thymidylate synthetase using 5-fluoro-2'-deoxyuridylate as an active site titrant. Biochemistry.

[3] Santi D.V., McHenry C.S., Sommer H. (1974). Mechanism of interaction of thymidylate synthetase with 5-fluorodeoxyuridylate. Biochemistry.

[4] Santi D.V., McHenry C.S., Raines R.T., Ivanetich K.M. (1987). Kinetics and thermodynamics of the interaction of 5-fluoro-2'-deoxyuridylate with thymidylate synthase. Biochemistry.

[5] Ullman B., Lee M., Martin D.W., Santi D.V. (1978). Cytotoxicity of 5-fluoro-2'-deoxyuridine: requirement for reduced folate cofactors and antagonism by methotrexate. Proc. Natl. Acad. Sci. U. S. A..

[6] Machover D. (1997). A comprehensive review of 5-fluorouracil and leucovorin in patients with metastatic colorectal carcinoma. Cancer.

[7] Schekman R., Weiner J.H., Weiner A., Kornberg A. (1975). Ten proteins required for conversion of phiX174 single-stranded DNA to duplex form *in vitro*. Resolution reconstitution. J. Biol. Chem..

[8] Wickner W., Kornberg A. (1973). DNA polymerase 3 star requires ATP to start synthesis on a primed DNA. Proc. Natl. Acad. Sci. U. S. A..

[9] Wickner W., Kornberg A. (1974). A holoenzyme form of deoxyribonucleic acid polymerase III. Isolation properties. J. Biol. Chem..

[10] Wickner W., Schekman R., Geider K., Kornberg A. (1973). A new form of DNA polymerase 3 and a copolymerase replicate a long, single-stranded primer-template. Proc. Natl. Acad. Sci. U. S. A..

[11] McHenry C., Kornberg A. (1977). DNA polymerase III holoenzyme of Escherichia coli. Purification and resolution into subunits. J. Biol. Chem..

[12] McHenry C.S., Crow W. (1979). DNA polymerase III of Escherichia coli. Purification and identification of subunits. J. Biol. Chem..

[13] DiFrancesco R., Bhatnagar S.K., Brown A., Bessman M.J. (1984). The interaction of DNA polymerase III and the product of the Escherichia coli mutator gene, mutD. J. Biol. Chem..

[14] McHenry C., Oberfelder R., Johanson K., Tomasiewicz H., Franden M., Kelly TaM R. (1987). Structure and Mechanism of the DNA Polymerase III Holoenzyme in DNA Replication and Recombination.

[15] McHenry C.S. (1982). Purification and characterization of DNA polymerase III'. Identification of tau as a subunit of the DNA polymerase III holoenzyme. J. Biol. Chem..

[16] Sinha N.K., Morris C.F., Alberts B.M. (1980). Efficient *in vitro* replication of double-stranded DNA templates by a purified T4 bacteriophage replication system. J. Biol. Chem..

[17] Johanson K.O., McHenry C.S. (1980). Purification and characterization of the beta subunit of the DNA polymerase III holoenzyme of Escherichia coli. J. Biol. Chem..

[18] Kornberg A. (1974). DNA Synthesis.

[19] Johanson K.O., McHenry C.S. (1982). The beta subunit of the DNA polymerase III holoenzyme becomes inaccessible to antibody after formation of an initiation complex with primed DNA. J. Biol. Chem..

[20] Fay P.J., Johanson K.O., McHenry C.S., Bambara R.A. (1981). Size classes of products synthesized processively by DNA polymerase III and DNA polymerase III holoenzyme of Escherichia coli. J. Biol. Chem..

[21] Crute J.J., LaDuca R.J., Johanson K.O., McHenry C.S., Bambara R.A. (1983). Excess beta subunit can bypass the ATP requirement for highly processive synthesis by the Escherichia coli DNA polymerase III holoenzyme. J. Biol. Chem..

[22] LaDuca R.J., Crute J.J., McHenry C.S., Bambara R.A. (1986). The beta subunit of the Escherichia coli DNA polymerase III holoenzyme interacts functionally with the catalytic core in the absence of other subunits. J. Biol. Chem..

[23] Kong X.P., Onrust R., O'Donnell M., Kuriyan J. (1992). Three-dimensional structure of the beta subunit of E. coli DNA polymerase III holoenzyme: a sliding DNA clamp. Cell.

[24] O'Donnell M., Kuriyan J., Kong X.P., Stukenberg P.T., Onrust R. (1992). The sliding clamp of DNA polymerase III holoenzyme encircles DNA. Mol. Biol. Cell.

[25] Huang C.C., Hearst J.E., Alberts B.M. (1981). Two types of replication proteins increase the rate at which T4 DNA polymerase traverses the helical regions in a single-stranded DNA template. J. Biol. Chem..

[26] Welch M.M., McHenry C.S. (1982). Cloning and identification of the product of the dnaE gene of Escherichia coli. J. Bacteriol..

[27] Tomasiewicz H.G., McHenry C.S. (1987). Sequence analysis of the Escherichia coli dnaE gene. J. Bacteriol..

[28] Flower A.M., McHenry C.S. (1986). The adjacent dnaZ and dnaX genes of Escherichia coli are contained within one continuous open reading frame. Nucleic Acids Res..

[29] Truitt C.L., Walker J.R. (1974). Growth of phages lambda, phiX174, and Ml3 requires the dnaZ (previously dnaH) gene product of Escherichia coli. Biochem. Biophys. Res. Commun..

[30] Henson J.M., Chu H., Irwin C.A., Walker J.R. (1979). Isolation and characterization of dnaX and dnaY temperature-sensitive mutants of Escherichia coli. Genetics.

[31] Lee S.H., Kanda P., Kennedy R.C., Walker J.R. (1987). Relation of the Escherichia coli dnaX gene to its two products--the tau and gamma subunits of DNA polymerase III holoenzyme. Nucleic Acids Res..

[32] McHenry C., Griep M., Tomasiewicz H., Bradley M. (1989). Molecular Mechanisms in DNA Replication and Recombination, Lehman CRaIR.

[33] Flower A.M., McHenry C.S. (1990). The gamma subunit of DNA polymerase III holoenzyme of Escherichia coli is produced by ribosomal frameshifting. Proc. Natl. Acad. Sci. U. S. A..

[34] Blinkowa A.L., Walker J.R. (1990). Programmed ribosomal frameshifting generates the Escherichia coli DNA polymerase III gamma subunit from within the tau subunit reading frame. Nucleic Acids Res..

[35] Tsuchihashi Z., Kornberg A. (1990). Translational frameshifting generates the gamma subunit of DNA polymerase III holoenzyme. Proc. Natl. Acad. Sci. U. S. A..

[36] Carter J.R., Franden M.A., Aebersold R., Kim D.R., McHenry C.S. (1993). Isolation, sequencing and overexpression of the gene encoding the theta subunit of DNA polymerase III holoenzyme. Nucleic Acids Res..

[37] Carter J.R., Franden M.A., Aebersold R., McHenry C.S. (1992). Molecular cloning, sequencing, and overexpression of the structural gene encoding the delta subunit of Escherichia coli DNA polymerase III holoenzyme. J. Bacteriol..

[38] Carter J.R., Franden M.A., Aebersold R., McHenry C.S. (1993). Identification, isolation, and overexpression of the gene encoding the psi subunit of DNA polymerase III holoenzyme. J. Bacteriol..

[39] Carter J.R., Franden M.A., Aebersold R., McHenry C.S. (1993). Identification, isolation, and characterization of the structural gene encoding the delta' subunit of Escherichia coli DNA polymerase III holoenzyme. J. Bacteriol..

[40] Carter J.R., Franden M.A., Lippincott J.A., McHenry C.S. (1993). Identification, molecular cloning and characterization of the gene encoding the chi subunit of DNA polymerase III holoenzyme of Escherichia coli. Mol. Gen. Genet..

[41] Dong Z., Onrust R., Skangalis M., O'Donnell M. (1993). DNA polymerase III accessory proteins. I. holA and holB encoding delta and delta'. J. Biol. Chem..

[42] Studwell-Vaughan P.S., O'Donnell M. (1993). DNA polymerase III accessory proteins. V. Theta encoded by holE. J. Biol. Chem..

[43] Xiao H., Crombie R., Dong Z., Onrust R., O'Donnell M. (1993). DNA polymerase III accessory proteins. III. holC and holD encoding chi and psi. J. Biol. Chem..

[44] Schatz P.J. (1993). Use of peptide libraries to map the substrate specificity of a peptide-modifying enzyme: a 13 residue consensus peptide specifies biotinylation in Escherichia coli. Biotechnology (N Y).

[45] Kim D.R., McHenry C.S. (1996). Biotin tagging deletion analysis of domain limits involved in protein-macromolecular interactions. Mapping the tau binding domain of the DNA polymerase III alpha subunit. J. Biol. Chem..

[46] Kim D.R., McHenry C.S. (1996). Identification of the beta-binding domain of the alpha subunit of Escherichia coli polymerase III holoenzyme. J. Biol. Chem..

[47] Wieczorek A., McHenry C.S. (2006). The NH2-terminal php domain of the alpha subunit of the Escherichia coli replicase binds the epsilon proofreading subunit. J. Biol. Chem..

[48] Gao D., McHenry C.S. (2001). Tau binds and organizes Escherichia coli replication proteins through distinct domains. Domain IV, located within the unique C terminus of tau, binds the replication fork, helicase, DnaB. J. Biol. Chem..

[49] Gao D., McHenry C.S. (2001). Tau binds and organizes Escherichia coli replication through distinct domains. Partial proteolysis of terminally tagged tau to determine candidate domains and to assign domain V as the alpha binding domain. J. Biol. Chem..

[50] Gao D., McHenry C.S. (2001). Tau binds and organizes Escherichia coli replication proteins through distinct domains. Domain III, shared by gamma and tau, binds delta delta ' and chi psi. J. Biol. Chem..

[51] Song M.S., McHenry C.S. (2001). Carboxyl-terminal domain III of the delta' subunit of DNA polymerase III holoenzyme binds DnaX and supports cooperative DnaX complex assembly. J. Biol. Chem..

[52] Bullard J.M., Pritchard A.E., Song M.S., Glover B.P., Wieczorek A., Chen J. (2002). A three-domain structure for the delta subunit of the DNA polymerase III holoenzyme delta domain III binds delta' and assembles into the DnaX complex. J. Biol. Chem..

[53] Glover B.P., Pritchard A.E., McHenry C.S. (2001). Tau binds and organizes Escherichia coli replication proteins through distinct domains: domain III, shared by gamma and tau, oligomerizes DnaX. J. Biol. Chem..

[54] Olson M.W., Dallmann H.G., McHenry C.S. (1995). DnaX complex of Escherichia coli DNA polymerase III holoenzyme. The chi psi complex functions by increasing the affinity of tau and gamma for delta.delta' to a physiologically relevant range. J. Biol. Chem..

[55] Aravind L., Koonin E.V. (1998). Phosphoesterase domains associated with DNA polymerases of diverse origins. Nucleic Acids Res..

[56] Stano N.M., Chen J., McHenry C.S. (2006). A coproofreading Zn(2+)-dependent exonuclease within a bacterial replicase. Nat. Struct. Mol. Biol..

[57] Johanson K.O., McHenry C.S. (1984). Adenosine 5'-O-(3-thiotriphosphate) can support the formation of an initiation complex between the DNA polymerase III holoenzyme and primed DNA. J. Biol. Chem..

[58] McHenry C.S., Johanson K.O. (1984). DNA polymerase III holoenzyme of Escherichia coli: an asymmetric dimeric replicative complex containing distinguishable leading and lagging strand polymerases. Adv. Exp. Med. Biol..

[59] Glover B.P., McHenry C.S. (2001). The DNA polymerase III holoenzyme: an asymmetric dimeric replicative complex with leading and lagging strand polymerases. Cell.

[60] Glover B.P., McHenry C.S. (2000). The DnaX-binding subunits delta' and psi are bound to gamma and not tau in the DNA polymerase III holoenzyme. J. Biol. Chem..

[61] Griep M.A., McHenry C.S. (1988). The dimer of the beta subunit of Escherichia coli DNA polymerase III holoenzyme is dissociated into monomers upon binding magnesium(II). Biochemistry.

[62] Griep M.A., McHenry C.S. (1992). Fluorescence energy transfer between the primer and the beta subunit of the DNA polymerase III holoenzyme. J. Biol. Chem..

[63] Reems J.A., Wood S., McHenry C.S. (1995). Escherichia coli DNA polymerase III holoenzyme subunits alpha, beta, and gamma directly contact the primer-template. J. Biol. Chem..

[64] Reems J.A., McHenry C.S. (1994). Escherichia coli DNA polymerase III holoenzyme footprints three helical turns of its primer. J. Biol. Chem..

[65] Dallmann H.G., McHenry C.S. (1995). DnaX complex of Escherichia coli DNA polymerase III holoenzyme. Physical characterization of the DnaX subunits and complexes. J. Biol. Chem..

[66] Pritchard A.E., Dallmann H.G., Glover B.P., McHenry C.S. (2000). A novel assembly mechanism for the DNA polymerase III holoenzyme DnaX complex: association of deltadelta' with DnaX(4) forms DnaX(3)deltadelta'. EMBO J..

[67] Onrust R., Finkelstein J., Turner J., Naktinis V., O'Donnell M. (1995). Assembly of a chromosomal replication machine: two DNA polymerases, a clamp loader, and sliding clamps in one holoenzyme particle. III. Interface between two polymerases and the clamp loader. J. Biol. Chem..

[68] Pritchard A.E., McHenry C.S. (2001). Assembly of DNA polymerase III holoenzyme: co-assembly of gamma and tau is inhibited by DnaX complex accessory proteins but stimulated by DNA polymerase III core. J. Biol. Chem..

[69] McHenry C.S. (2011). DNA replicases from a bacterial perspective. Annu. Rev. Biochem..

[70] Dallmann H.G., Fackelmayer O.J., Tomer G., Chen J., Wiktor-Becker A., Ferrara T. (2010). Parallel multiplicative target screening against divergent bacterial replicases: identification of specific inhibitors with broad spectrum potential. Biochemistry.

[71] Sanders G.M., Dallmann H.G., McHenry C.S. (2010). Reconstitution of the B. subtilis replisome with 13 proteins including two distinct replicases. Mol. Cell.

[72] Zinder J.C. (2013).

[73] Seco E.M., Zinder J.C., Manhart C.M., Lo Piano A., McHenry C.S., andAyora S. (2013). Bacteriophage SPP1 DNA replication strategies promote viral and disable host replication *in vitro*. Nucleic Acids Res..

[74] Downey C.D., McHenry C.S. (2010). Chaperoning of a replicative polymerase onto a newly assembled DNA-bound sliding clamp by the clamp loader. Mol. Cell.

[75] Dalrymple B.P., Kongsuwan K., Wijffels G., Dixon N.E., Jennings P.A. (2001). A universal protein-protein interaction motif in the eubacterial DNA replication and repair systems. Proc. Natl. Acad. Sci. U. S. A..

[76] Lopez de Saro F.J., Georgescu R.E., O'Donnell M. (2003). A peptide switch regulates DNA polymerase processivity. Proc. Natl. Acad. Sci. U. S. A..

[77] Leu F.P., Georgescu R., O'Donnell M. (2003). Mechanism of the E. coli tau processivity switch during lagging-strand synthesis. Mol. Cell.

[78] Lopez de Saro F.J., Georgescu R.E., Goodman M.F., O'Donnell M. (2003). Competitive processivity-clamp usage by DNA polymerases during DNA replication and repair. EMBO J..

[79] Dohrmann P.R., McHenry C.S. (2005). A bipartite polymerase-processivity factor interaction: only the internal beta binding site of the alpha subunit is required for processive replication by the DNA polymerase III holoenzyme. J. Mol. Biol..

[80] Liu B., Lin J., Steitz T.A. (2013). Structure of the PolIIIalpha-tauc-DNA complex suggests an atomic model of the replisome. Structure.

[81] Hawker J.R., McHenry C.S. (1987). Monoclonal antibodies specific for the tau subunit of the DNA polymerase III holoenzyme of Escherichia coli. Use to demonstrate that tau is the product of the dnaZX gene and that both it and gamma, the dnaZ gene product, are integral components of the same enzyme assembly. J. Biol. Chem..

[82] McInerney P., Johnson A., Katz F., O'Donnell M. (2007). Characterization of a triple DNA polymerase replisome. Mol. Cell.

[83] Reyes-Lamothe R., Sherratt D.J., Leake M.C. (2010). Stoichiometry and architecture of active DNA replication machinery in Escherichia coli. Science.

[84] Dohrmann P.R., Correa R., Frisch R.L., Rosenberg S.M., McHenry C.S. (2016). The DNA polymerase III holoenzyme contains gamma and is not a trimeric polymerase. Nucleic Acids Res..

[85] Yuan Q., McHenry C.S. (2014). Cycling of the E. coli lagging strand polymerase is triggered exclusively by the availability of a new primer at the replication fork. Nucleic Acids Res..

[86] Downey C.D., Crooke E., McHenry C.S. (2011). Polymerase chaperoning and multiple ATPase sites enable the E. coli DNA polymerase III holoenzyme to rapidly form initiation complexes. J. Mol. Biol..

[87] Yuan Q., Dohrmann P.R., Sutton M.D., McHenry C.S. (2016). DNA polymerase III, but not polymerase IV, must Be bound to a tau-containing DnaX complex to enable exchange into replication forks. J. Biol. Chem..

[88] Lindow J.C., Dohrmann P.R., McHenry C.S. (2015). DNA polymerase alpha subunit residues and interactions required for efficient initiation complex formation identified by a genetic selection. J. Biol. Chem..

[89] Wu C.A., Zechner E.L., Hughes A.J., Franden M.A., McHenry C.S., Marians K.J. (1992). Coordinated leading- and lagging-strand synthesis at the Escherichia coli DNA replication fork. IV. Reconstitution of an asymmetric, dimeric DNA polymerase III holoenzyme. J. Biol. Chem..

[90] Wu C.A., Zechner E.L., Reems J.A., McHenry C.S., Marians K.J. (1992). Coordinated leading- and lagging-strand synthesis at the Escherichia coli DNA replication fork. V. Primase action regulates the cycle of Okazaki fragment synthesis. J. Biol. Chem..

[91] Kim S., Dallmann H.G., McHenry C.S., Marians K.J. (1996). Coupling of a replicative polymerase and helicase: a tau-DnaB interaction mediates rapid replication fork movement. Cell.

[92] Manhart C.M., McHenry C.S. (2013). The PriA replication restart protein blocks replicase access prior to helicase assembly and directs template specificity through its ATPase activity. J. Biol. Chem..

[93] Yuan Q., McHenry C.S. (2009). Strand displacement by DNA polymerase III occurs through a tau-psi-chi link to single-stranded DNA-binding protein coating the lagging strand template. J. Biol. Chem..

[94] Dohrmann P.R., Manhart C.M., Downey C.D., McHenry C.S. (2011). The rate of polymerase release upon filling the gap between Okazaki fragments is inadequate to support cycling during lagging strand synthesis. J. Mol. Biol..

[95] Lee J., Chastain P.D., Kusakabe T., Griffith J.D., Richardson C.C. (1998). Coordinated leading and lagging strand DNA synthesis on a minicircular template. Mol. Cell.

[96] Yang J., Nelson S.W., Benkovic S.J. (2006). The control mechanism for lagging strand polymerase recycling during bacteriophage T4 DNA replication. Mol. Cell.

[97] Li X., Marians K.J. (2000). Two distinct triggers for cycling of the lagging strand polymerase at the replication fork. J. Biol. Chem..

[98] Jergic S., Horan N.P., Elshenawy M.M., Mason C.E., Urathamakul T., Ozawa K. (2013). A direct proofreader-clamp interaction stabilizes the Pol III replicase in the polymerization mode. EMBO J..

